# The tumor microenvironment in pancreatic cancer: from composition to therapeutic targeting

**DOI:** 10.1042/BST20260001

**Published:** 2026-08-07

**Authors:** Beatriz Magalhães Gomes, Pedro Luiz Porfirio Xavier

**Affiliations:** Laboratory of Comparative and Translational Oncology, Department of Veterinary Medicine, School of Animal Science and Food Engineering, Universidade de São Paulo (USP), Pirassununga, Brazil

**Keywords:** Cancer-associated fibroblasts, extracellular matrix, Pancreatic Ductal Adenocarcinoma, tumor microenvironments

## Abstract

Pancreatic cancer, predominantly represented by Pancreatic Ductal Adenocarcinoma (PDAC), is one of the most frequent and deadly types of cancer. In contrast with other types of cancer, for which advances in prevention, early detection, and treatment have contributed to decreasing incidence and mortality, PDAC continues to be rarely diagnosed at early stages of the disease and exhibits a poor prognosis. As a consequence, therapeutic efficacy remains limited, which is reflected in high mortality. Therefore, the development of novel and effective therapeutic strategies against PDAC is urgent. However, a major component limiting therapeutic efficacy is the highly complex PDAC tumor microenvironment (TME), which is composed of cancer-associated fibroblasts, immunosuppressive cells, cancer stem cells, and a dense extracellular matrix, a structural and biochemical scaffold that sustains tumor development and influences several PDAC phenotypes including metabolism, immune infiltration, metastasis, and therapeutic response. The present review focuses on and discusses the key components of the PDAC TME, with particular emphasis on ECM remodeling, stromal components, their impact on tumor progression and therapeutic resistance, and emerging strategies to target these processes.

## Introduction

Pancreatic cancer is one of the deadliest types of cancer, with a 5-year survival rate lower than 13% [1,2]. While advances in early diagnosis and therapies have improved the outcome for other types of cancer, Pancreatic Ductal Adenocarcinoma (PDAC) remains a major challenge, with patients usually diagnosed at a late stage of disease. Surgical resection is the main therapeutic option. However, it is limited to patients with localized and surgically resectable disease. For most patients with regional or distant metastasis, chemotherapy is the mainstay of PDAC treatment, still resulting in low efficacy and survival rates [1,3–5]. Therefore, identifying novel and effective therapies against PDAC is crucial. Nevertheless, this objective faces a major obstacle: the PDAC tumor microenvironment (TME). The PDAC TME is a highly complex cellular and stromal component that exerts paradoxical roles in PDAC progression. From one perspective, the dense fibrotic stroma surrounding cancer cells can act as a physical barrier, restraining tumor development and dissemination. In contrast, desmoplasia also creates a mechanical barrier that limits drug delivery and immune cell infiltration, while impairing vascularization. These phenotypes are important for a hypoxic and more aggressive TME.

Herein, we will discuss the complexity of the PDAC TME, stroma-cancer cells interactions, and the key role of TME in tumor development and treatment resistance. We also focus on providing therapeutic strategies to target TME and overcome this barrier in combination with other therapies.

## Extracellular matrix: composition and properties

The PDAC TME is surrounded by immune cells, endothelial cells, and a wide variety of fibroblasts, presenting a well-established relevance for PDAC progression. Moreover, it is characterized by a prominent desmoplastic reaction in which cancer cells activate cancer-associated fibroblasts (CAFs) to induce fibrosis and deposition of extracellular matrix (ECM) [6,7]. The ECM is composed of collagen, hyaluronan, glycoproteins, proteoglycans, fibronectin, enzymes, growth factors, and chemokines that offer physical support and biochemical signals for cancer cellular populations [8,9]. Consistently, ECM-rich/desmoplastic transcriptional signatures are linked to poor prognosis in PDAC [10,11]. Recently, by integrating multi-OMICs, clinical data, and patient-derived preclinical models, it was demonstrated that PDAC TME is organized into spatially confined subTMEs. The ‘deserted’ TME is characterized by abundant ECM deposition, tumor-suppressive features, and strong chemoprotective properties. ‘Reactive’ TME is rich in complex and active fibroblasts, vascularized, with inflammatory signaling and immune infiltration, promoting tumor proliferation and basal-like phenotypes. Interestingly, PDAC tumors exhibiting intermediate levels and co-occurrence of intratumoral subTMEs were associated with poorer prognosis, linking stromal heterogeneity to patient outcome and suggesting that these tumors may benefit from tumor-promoting properties of reactive subTMEs and chemoprotective properties of deserted subTMEs [12].

**Figure 1:**
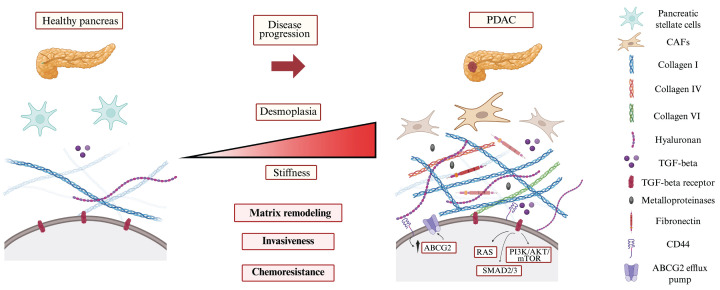
The remodeling and composition of ECM in PDAC ECM remodeling in PDAC is characterized by progressive changes in the abundance, composition, organization, and complexity of matrisome proteins during the transition from a healthy pancreas to malignant PDAC. This process involves increased deposition of structural ECM components, such as collagens, and enhanced activity of ECM-modifying enzymes, including hyaluronidases and matrix metalloproteinases (MMPs). Furthermore, tumor-promoting signaling pathways, such as TGF-β signaling, contribute to stromal activation, matrix remodeling, and desmoplasia. The remodeled ECM also supports the activation of cancer-associated pathways, including RAS, PI3K/AKT/mTOR, and ABCG2 (ATP-binding cassette sub-family G member 2)-related signaling, influencing tumor progression, therapeutic resistance, and the aggressiveness of the PDAC microenvironment.

ECM remodeling dynamically occurs throughout PDAC progression, influencing several PDAC phenotypes (Figure 1). ECM remodeling exhibits progressive changes in abundance, composition, and complexity of matrisome proteins from normal pancreas to pancreatic intraepithelial neoplasia (PanINs) and, ultimately, PDAC [9]. Furthermore, recent evidence has demonstrated that ECM remodeling and organization occur during the early stages of PDAC progression and are linked to early PDAC invasion [13]. ECM remodeling also directly impacts metabolism and therapeutic responses. The degradation of hyaluronan, for instance, directly promotes glucose metabolism through ZFP36-TXNIP-GLUT1 signaling, resulting in enhanced glycolytic activity [14]. More recently, it was demonstrated that ECM remodeling promotes the production and accumulation of guanylates, guanine-derived nucleotides that support DNA synthesis and repair, protecting PDAC cells from oxaliplatin-induced DNA damage [15].

Collagen, especially type I (Col1), is the most abundant ECM component across all stages of the disease and is not merely a structural component of desmoplasia [9]. Importantly, a plethora of studies have demonstrated a dichotomous role of Col1 in PDAC. Col1 is frequently linked to tumorigenesis, enhanced cellular proliferation, evasion of the immune system, migration, and overall survival [16]. For instance, patients with high levels of Col1 had a median survival of 6.4 months, whereas patients with low Col1 had an overall survival of 14.6 months [17]. However, the cellular origin of Col1 appears to influence PDAC progression and development. Col1 produced by PDAC cells differs from that produced by CAFs due to the formation of abnormal collagen 1A1 homotrimers. These homotrimers promote cancer cell growth and proliferation by binding to α3β1 integrin on cancer cells [18]. In contrast, myofibroblast-specific deletion of Col1 accelerates the emergence of PanINs and PDAC progression, reducing overall survival in mouse models [19]. Furthermore, the structural integrity of Col1 can also modify its biological activity. MMP-cleaved Col1 promotes PDAC growth mediated by activation of DDR1-NF-kB-p62-NRF2 signaling, decreasing patient survival. Conversely, intact Col1 induces the degradation of DDR1 and restrains the growth of PDAC, increasing patients’ survival [20].

Other collagens also emerge in PDAC biology. Fibrillar collagens undergo abnormal processing during PDAC progression, including the increased retention of the C-terminal propeptide of Col3A1 [21]. In inflammatory and CAF-driven niches, Col3A1 may be related to promoting metastasis, stromal remodeling, and immune exclusion [22]. Also, Col3 turnover in plasma may be considered a blood-based predictive biomarker in metastatic PDAC patients and a molecular biomarker for prognosis and immune infiltration [23,24]. As PDAC progresses, Col6A3 and Col4 become increasingly abundant and have been associated with metastasis to soft tissues, particularly to the liver [25,26]. The remodeling of collagens in PDAC can be so dynamic that even therapies are being shown to remodel the collagen profile. Neoadjuvant therapies were demonstrated to decrease type I, III, IV, and V collagen by reducing the number of ephrin-A5^+^ CAFs [26–28]. Collectively, these studies highlight the relevance of collagens within PDAC ECM and demonstrate their tumor-promoting effects.

Additional ECM components have crucial roles for PDAC. Patients with high levels of hyaluronan, for example, have a lower median survival and response to chemotherapies [17,29]. ECM proteins produced by malignant cells, including agrin, serine protease inhibitor B5, and cystatin B, are intrinsically associated with low survival rates [30]. Meanwhile, ECM factors produced by stromal cells have a dual role, being positively or negatively correlated to patients’ survival [9,30]. Regarding glycoproteins, fibrillin 1, fibronectin (FN1), fibrinogens FGA, FGB, and FGG, and periostin are the most abundant in the PDAC ECM. The majority of these molecules are also produced by stromal cells, performing key roles in PDAC phenotypes [9]. FN1 is deposited by cancer cells in the lack of cell–cell contact through the up-regulation of lysophosphatidic acid receptor 4. Thus, FN1 interacts with integrins such as α5β1 and αvβ3, supporting growth and stemness phenotypes [31]. As well, FN1 derived from CAFs interacts with integrin receptors and promotes migration and invasion via integrin PI3K-AKT signaling [32]. Recently, other glycoproteins have also been implicated in the modulation of important therapy-response phenotypes. Prosaposin, a secreted glycoprotein that regulates lysosomal sphingolipid, has been demonstrated to modulate the infiltration of immune therapy, suppressing the infiltration of CD8^+^ T tumor-infiltrating lymphocytes [33].

The main factor regulating ECM proteins is TGF-β1 (transforming growth factor-β), produced by cancer and stromal cells [9]. TGF-β represents a subfamily of cytokines with a dual role in cancer biology, exhibiting both pro- and anti-tumor roles [34,35]. In PDAC early stages, TGF-β has been demonstrated to play a role in tumoral suppression [36]. However, as PDAC progresses and becomes more aggressive, TGF-β becomes associated with oncogenic cellular pathways, such as PI3K/AKT/mTOR, Ras, and SMAD2/3, regulating tumoral growth, cytoskeleton remodeling, epithelial-mesenchymal transition (EMT), perineural invasion, desmoplasia, inflammation, immunosuppressive microenvironment, and metastasis. These processes are linked with the activation of the transcription factors, including ZEB1/ZEB2, SNAIL, and SLUG [32,35–42]. TGF-β also regulates the enrichment of cancer stem cells (CSCs) in the population and promotes the acquisition of chemotherapy resistance [43].

The PDAC stiffness is fundamental to progression and therapy response. Stiffness is directly associated with ECM remodeling, deposition, and disease progression, activating cell signaling and molecular pathways related to malignant phenotype and acquisition of chemotherapy resistance [44–46]. The progression toward a stiffer environment, it is illustrated by comparing the Young’s modulus of healthy pancreatic tissue and PDAC. While normal pancreatic tissue has a Young’s modulus of 2.2 kPa (kilopascal), PDAC reaches a modulus of 7.4 kPa, indicating an increase in rigidity and stiffness [47]. This modulates the transcriptomic profile and up-regulates metalloproteinases, such as MMP3, genes related to cell motility and ECM factors, including Col1A1 and FN1 [47]. Membrane potential, which can be altered by tissue density and mechanical pressure, acts as a regulator of YAP (Yes-associated protein), an important mechanosensitive protein [48] related to acinar-ductal metaplasia, tumorigenesis, stemness, lipid metabolism, cell proliferation, activation of fibroblasts, fibrosis, and chemoresistance [48–53]. Given the relevance of stiffness in PDAC progression, more reliable and complex cell culture models integrating stiffness as a major component have been developed [47,54–58]. Taken together, these studies highlight the complexity of the ECM and reveal dynamic mechanisms that shape PDAC tumor progression and modulate therapy resistance.

## Cell populations in PDAC stroma: cancer-associated fibroblasts

CAFs are highly heterogeneous and play a key role in PDAC ECM remodeling, tumor development, progression, and metastasis [59–63]. CAFs are mainly derived from pancreatic stellate cells (PSCs) [64,65] but can also be derived from stellate hepatic cells, mesenchymal stem cells, endothelial cells, adipocytes, resident fibroblasts, pericytes, epithelial cells, and cancer-stem cells [66,67]. In general, three subpopulations of CAFs are established, based on the expression of distinct markers. Myofibroblasts (myCAFs) exhibit high α-SMA, periostin, CTGF, and TGF-β pathway activity [68,69]. myCAFs are located adjacent to malignant cells and are the principal producers of fibrillar collagen and crosslinking enzymes, including LOX/LOXL2 and PLOD2/3, contributing to increasing ECM stiffness and the architecture of PDAC [70]. Inflammatory CAFs (iCAFs) are characterized by the secretion of cytokines and chemokines, including LIF, IL-6, IL-11, and CXCL12, and are located at a distance from malignant cells [59,61,68,71–73]. iCAFs can be implicated in matrix turnover, inducing matrix metalloproteinases, favoring degradation and remodeling [71]. Finally, antigen-presenting fibroblasts (apCAFs) are the rarest CAF population, characterized by MHC-II and CD74 expression and immunomodulatory roles [74].

However, advances in single-cell transcriptomics have enabled and proposed nine different CAF subtypes, revealing newly functional roles within TME (Table 1) [69]. Wang et al. proposed a novel metabolic state CAF (meCAFs), responsible for supplying intermediate metabolites to PDAC cells, supporting glycolysis in hypoxic environments [75]. Senescent CAFs, which are maintained in a cell-cycle arrest state, contribute to tumor proliferation, progression, and immunosuppression through the secretion of plasminogen activator urokinase [76,77]. Tumor-like CAFs (tCAFs) were recently described as promoters of EMT and ECM stiffness [52]. Reticular CAFs (rCAFs) are intrinsically related to the formation of tertiary lymphoid structures and the infiltration of T lymphocytes in the tumor [78]. Therefore, many new subtypes of CAFs are being described, highlighting the importance of these cells in the TME.

**Table 1:** Cancer-associated fibroblast subtypes and their representative markers in pancreatic ductal adenocarcinoma

CAF subtypes	Markers	Reference
myCAFs	Overexpression of TGFB1, SMAD2, TWIST1 and PDFGRL	[69]
	α-SMA, ACTA2, CTGF	[59]
	PDPN+, Ly6c-, MHC-II-	[78]
iCAFs	LIF, IL-1a, IL-6, CXCL1, CSF3	[59]
	Overexpression of CXCL2, CXCL12, CCL2, FOS, JUNB, JUND, GFG7, genes of JAK-STAT pathway	[69]
	PDPN+, Ly6c+, MHC-II-	[78]
apCAFs	MHC-II and CD74	[74]
	Overexpression of CF75 and HLA genes	[69]
	Ly6c -, MHC-II+, CD74+	[78]
ISG+ myCAF: interferon stimulated genes myCAFs	Overexpression of ISGs, including ISG15 and IFIT1	[69]
tCAF: transitional CAF	Overexpression of CST2, ANOX1, FOXS1 and HOPX	[69]
nCAF: normal-like fibroblasts	Overexpression of CFD, PDGFRA and CXCL12	[69]
pCAF: proliferative CAFs	Overexpression of MK167 and TOP2A	[69]
TSPAN8+ CAF: membrane protein tetraspanin 8+ CAF	TSPAN8, overexpression of FXYD3, TFF1 and TFF3	[69]
CDCP1 + FTL + CAF: CUB domain containing protein 1+ Ferritin light chain+ CAFs	FTL, overexpression of FTH1, ENO1 and PGK1	[69]
tCAF: tumor-like CAF	CD10, CD73 and LOXL2 positive	[52]
mCAF: matrix-associated CAF	TGM2 positive	[52]
meCAF: metabolic state CAF	Overexpression of PLA2G2A and CRABP2	[75]
sCAF: senescent CAF	H-SCAF: MIF, CXCL12, VEGF	[76]
rCAF: reticular CAF	ICAM+, VCAM-1+. Expression of CCL19, CCL21	[78]

Recent studies demonstrate that CAF differentiation and plasticity have a key role in PDAC ECM architecture. For instance, scRNA-seq identified a novel CAF subset, tCAFs, enriched in tumors with high YAP1 activity. YAP1 mediates the conversion of mCAFs to tCAFs and, consequently, remodels ECM, increases stiffness, and is related to lower survival. In addition, disruption of the PSC myofibroblastic reduces myCAF features, including α-SMA expression and contractility, but paradoxically enhances pancreatic cancer invasion. These findings suggest CAFs are not uniformly tumor-promoting [52,65]. Tao et al. considered transitional CAFs as an intermediate state between iCAFs and myCAFs. When treated with TGF-β, CAFs were reprogrammed toward a myCAFs transcriptional profile. Meanwhile, treatment with IFN-γ, a pro-inflammatory cytokine, led to an iCAFs transcriptional profile [69]. The inhibition of macropinocytosis is implicated in an increase in iCAFs and a decrease in myCAFs [79]. PDAC cells are capable of recruiting, activating, and educating CAFs to establish a more permissive state for metastasis. In return, CAFs promote PDAC cells’ migration through the production and deposition of perlecan (or HSPG2—heparan sulfate proteoglycan 2) in the ECM [80–82]. Therefore, these studies highlight CAFs’ plasticity as a key determinant of ECM remodeling and PDAC progression.

## Immune landscape

PDAC is characterized as an ‘immunologically cold tumor’ due to a highly immunosuppressive environment. The PDAC immune landscape includes myeloid cells, such as tumor-associated macrophages, especially the pro-tumor subtype M2, myeloid-derived suppressor cells (MDSC), neutrophils, natural killer cells, B lymphocytes, CD4^+^ T lymphocytes, regulatory lymphocytes (Tregs), Th17, and effector CD8^+^ T lymphocytes [8,69,83–85]. However, anti-tumor effector immune cells, such as CD8^+^ T cells, are often characterized by cellular exhaustion markers expression [8,69,83,85]. Recently, several studies have demonstrated the mechanisms behind PDAC immunosuppression. For instance, the production of cytokines, like GM-CSF (granulocyte-macrophage colony-stimulating factor), promotes the recruitment of immature MDSC cells to the tumor, preventing the proliferation of effector T lymphocytes [84,86,87]. In addition, cholesterol deficiency has been linked to exhaustion and autophagy of CD8^+^ T lymphocytes [88].

Tumor-associated neutrophils (TANs) are considered pivotal regulators in PDAC progression. Circulating neutrophils are recruited to the PDAC TME driven by chemokines and cytokines and converted to TANs, which acquire longevity and immunosuppressive phenotypes [89]. TANs were categorized into anti-tumorigenic (N1) and pro-tumorigenic (N2), based on the differential expression of ICAM-1, CD95, and CXCR2 [90]. However, scRNA-seq technology is demonstrating complex TAN heterogeneity in PDAC TME [91,92]. Nowadays, at least four subsets are described: a pro-tumorigenic subset (TAN-1), an inflammatory subset (TAN-2), a migratory subset (TAN-3), and an interferon-stimulated gene high subset (TAN-4) [91]. Regarding the pro-tumorigenic functions of TANs, the capacity of neutrophil extracellular trap (NET) formation has emerged as critical. NETs are structures composed of chromatin and chromatin-bound networks, granular proteins including histones, myeloperoxidase, and elastase, with antimicrobial potency [93]. NETs can promote PDAC progression through epithelial-mesenchymal transition, ECM remodeling, metastasis, and therapy resistance [94–98]. In addition, several studies have demonstrated the synergistic network between TANs and TME cellular components, including CAFs, macrophages, NK cells, and effector T cells and Tregs [99–102]. Collectively, the emerging evidence determines tumor-TANs as key orchestrators of PDAC progression.

Emerging evidence has also highlighted the contribution of CAFs to the PDAC immune landscape. Through the sialylation process, increased expression of sialic acids on the cell surface of both cancer cells and CAFs promotes immunosuppression via the connection of Siglec receptors expressed on NK cells, T lymphocytes, and TAMs [103,104]. Also, through the production of cytokines associated with the CXCL12-CXCR4 axis, CAFs stimulate chemotaxis of neutrophils and macrophages toward the tumor, a process correlated with poorer prognosis [69]. iCAFs can stimulate the differentiation of tumor-infiltrating monocytes into immunosuppressive macrophages [103–105]. Finally, apCAFs interact with CD4^+^ T lymphocytes inducing the formation of Tregs, which suppress the proliferation of CD8^+^ T cells and contribute to immune evasion [74].

Different strategies of immunotherapy have been intensively investigated in PDAC. A phase I/II clinical trial demonstrated that T helper lymphocytes expressing tumor-associated antigens (TAAs), such as survivin, were infused in patients with pancreatic cancer [106]. Clinical responses were observed, especially in the group of patients who had previously received chemotherapy. In addition, TAA-expressing T cells were able to expand and persist within the immunological system [106]. An immunotoxin targeting mesothelin (LMB-100), a protein on the surface of cells, was found to modulate the PDAC TME by increasing the infiltration of CD4^+^ and CD8^+^ T lymphocytes in pancreatic tumors [107]. Targeting the CXCR4-CXCL12 axis showed mild effects [108]. A recent clinical trial that combined the agonists of CD137 T lymphocytes, anti-PD1 (programmed death receptor), and a GM-CSF-secreting vaccine, called GVAX, showed modulation of the immune landscape with reduction of immunosuppressive Tregs and an increase in active CD8^+^ T lymphocytes [109,110]. Interestingly, genes differentially expressed between patients treated with GVAX alone and those receiving combination therapies included ECM-related genes, such as FN1, cytoskeleton genes, and markers of T cell exhaustion. Nevertheless, TAMs were also found to undergo cytoskeletal and phenotypical changes, especially regarding the TREM2 signaling pathway, which could lead to immune evasion and the development of resistance [109].

## Peripheral nervous system: infiltrating neurons

The peripheral nervous system has emerged as a novel area in cancer biology due to growing evidence of its involvement in tumor progression and immunosuppression. A signaling axis between the peripheral system and lung adenocarcinoma was identified. Cancer cells can produce factors that promote innervation and activation of vagal sensory neurons, which, through cell–cell cross-talk, activate immunosuppressive macrophages and inhibit T-cell tumor responses [111]. In PDAC, tumor cells were found to reprogram neurons to interact with CAFs, thereby stimulating immunosuppression and tumor progression [112,113]. The role of CAFs in PDAC neural remodeling has also been investigated. CAFs were found to induce perineural invasion (PNI) by the release of extracellular vesicles containing PNI-associated transcript [114]. Furthermore, TGF-β-activated myofibroblasts were demonstrated to recruit sympathetic nerves to PanINs, who in turn released norepinephrine (NE). NE triggers desmoplasia, pro-inflammatory programs, and acinar-to-ductal metaplasia, demonstrating that neuro-stromal interactions promote PDAC progression [115]. Sympathetic signaling can also enhance CAFs activation and ECM remodeling, whereas activated CAFs promote transcriptional signaling associated with nerve injury responses [116]. Together, these findings highlight the key cross-talk between CAFs and sympathetic nerves, promoting neural remodeling and PDAC progression.

Sympathetic nerves were identified as a source of catecholamines inside the tumor, which promotes the up-regulation of the beta-adrenergic receptor adrenoceptor beta 1 and consequent exhaustion of CD8^+^ T cells [117]. In addition, sensory neurons were a source of glutamate for PDAC cells via an axis called neuro-cancer pseudo synapses, stimulating tumor growth and metastatic potential [118]. Glutamate produced by cancer cells also promoted metabolic reprogramming toward a glycolytic profile and PNI. Interestingly, PDAC cells were found capable of regulating the release of glutamate from neurons through the IGF1/TRPV1-PI3K/AKT axis [119]. Therefore, therapies targeting the interaction between PDAC and neuron cells emerge as a novel approach that holds great therapeutic potential [120].

## Cancer stem cells

CSCs have the ability of self-renewal, differentiation, and stemness [121], being related to several PDAC phenotypes [122,123]. Nowadays, CSCs are also considered a cell ‘state,’ since plasticity can induce tumor cells to acquire CSC properties [124]. The regulation of the CSC state can occur by PDAC ECM stimuli and composition. Type I Collagen activates FAK kinase (focal adhesion kinase), providing enhanced clonogenic cell growth and self-renewal of PDAC cells [125]. The deregulation of cell signaling pathways and epigenetic pathways can also lead to the acquisition of stemness [126], which can also be stimulated by CAFs [127]. In addition, fibroblasts can be transformed and acquire stemness characteristics through the absorption of free chromatin particles that are released in the extracellular environment after cancer cells death [128]. Immune cells also contribute to the activation of stemness-related pathways. For instance, TAMs were identified as activators of CSCs state in a co-culture system with PDAC cells, noted by the enhanced expression of stem cell genes, including OCT4, Nanog, and SOX2 [123,129].

CSCs are intrinsically related to therapeutic resistance [130,131]. Recently, a study identified a correlation between the expression of CSC markers with FOXP1, a gene that is strongly correlated with the pump efflux gene ABCG2 [132]. Beyond this, CSCs can survive even when chemotherapeutic approaches show efficiency on PDAC cancer cells, via the WNT-β-catenin and E2F-RB axis [133]. Exosomes containing the microRNA miR-210, derived from CSCs, were found to induce polarization of M2 macrophages, leading to gemcitabine resistance [134]. Taken together, these studies show the importance of targeting CSCs in order to induce PDAC to chemosensitivity.

## TME and metabolism reprogramming

ECM remodeling and the desmoplastic stroma create a unique metabolism for PDAC. Thus, several studies are investigating metabolism alterations and targets intrinsic to PDAC. For example, the cleaved state of collagen 1 leads to the activation of collagen receptor DDR1, triggering the axis DDR1-NF-κβ-p62-NFR2. This pathway enhances macropinocytosis and the production of mitochondria, elevating the metabolic and energetic potential of malignant cells [20]. Moreover, stromal cells were found to use amino acids as a substrate for energy production, like glutamine [58]. PDAC cells can use glutamine to support proliferation, redox balance, and ECM remodeling [135]. For instance, a recent study demonstrated that glutamine depletion and micropinocytosis inhibition can activate an inflammatory program in CAFs, promoting a transition from myCAFs to iCAFs and consequently altering PDAC stroma [79]. These studies opened an avenue to target glutamine metabolism in PDAC, and glutamine antagonists have presented promising effects in different models [136,137].

CAFs and cancer cells interact by metabolic exchanges to favor tumor development. PDAC cells stimulate CAFs to secrete extracellular vesicles containing enzymes and factors to avoid apoptosis due to a lack of nutrients [138,139]. PDAC cells, through aerobic glycolysis, produce lactic acid, which is converted into lactate and exported to the extracellular environment. This lactate accumulation promotes the differentiation of mesenchymal stem cells into CAFs through epigenetic modulation. Consequently, PDAC cells are able to influence the heterogeneity of TME [140]. Reciprocally, CAFs produce lactate that is absorbed by PDAC cells, triggering epigenetic changes through lactylation of histones, leading to PNI, metastasis, and poor prognosis [63].

Lipid metabolism, essential for cell viability, is also being explored. The lipid kinase PIKfyve is lacking in the healthy pancreas but is overexpressed in PanIN. In PDAC, PIKfyve is essential to progression. Interestingly, PIKfyve inhibition forces PDAC to up-regulate different transcriptional and metabolic programs that are usually activated by the KRAS-MAPK signaling pathway. Thus, combinatorial PIKfyve and KRAS-MAPK inhibition resulted in the elimination of PDAC models [141]. In addition, hypoxia caused by dense PDAC stroma leads to the interruption of unsaturated lipids production, which increases saturated lipids in hypoxic cells [142]. The availability of unsaturated lipids is essential for the viability of cancer cells. Therefore, it was found that myCAFs, owing to their spatial proximity to blood vessels, do not exhibit hypoxia and are able to supply lysophosphatidylcholine-derived unsaturated fatty acids to PDAC cells promoting their survival under hypoxic conditions [142]. Under metabolic stress, CAFs can also perform macropinocytosis, uptaking, degrading, and using serum albumin as a source of amino acids that can be excreted to support tumor growth [79]. Lipid supplementation of oleic acid was found to stimulate chemoresistance of PDAC cells to gemcitabine. Furthermore, lipid storage was found to be linked to stemness, since it serves as a source of energy to sustain the self-renewal and proliferation of CSCs [143].

## Clinical strategies and promise ECM therapeutic targets

**Figure 2:**
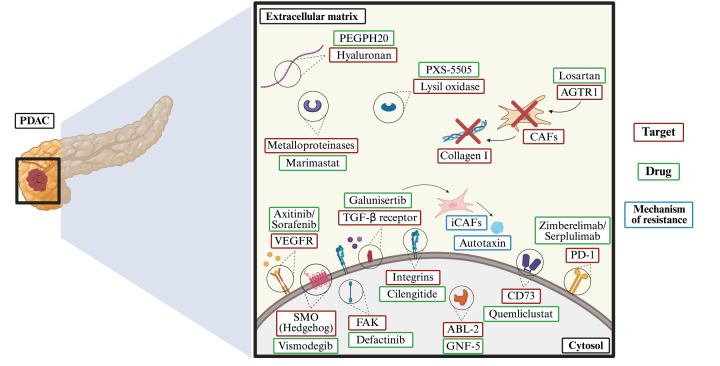
ECM-associated therapeutic targets in PDAC ECM-associated components and signaling pathways explored as therapeutic targets in preclinical models and clinical trials. The dense and remodeled ECM of PDAC contributes to desmoplasia, increased tissue stiffness, impaired drug delivery, immune exclusion, and therapy resistance. Several strategies have been investigated to target and reprogram this stromal compartment, including approaches directed against hyaluronan metabolism, collagen, TGF-β signaling, focal adhesion pathways, and other ECM-related mechanisms.

One of the first therapeutic strategies targeting PDAC TME failed. The depletion of desmoplasia via Sonic hedgehog inhibition increased tumor aggressiveness in mouse models, highlighting a deeper understanding of the stroma and the investigation of novel ECM targets [144]. More recently, several drugs targeting ECM components have been tested in clinical trials, with generally poor outcomes (Figure 2). For example, the Hedgehog inhibitor Vismodegib, combined with chemotherapy, failed to improve compared with chemotherapy alone [145] (NCT01088815, NCT01195415). Losartan, which inhibits type I collagen production by CAFs, provided no additional benefit when combined with FOLFIRINOX [146,147] (NCT01821729, NCT03563248). VEGFR inhibitors evaluated in clinical trials likewise failed to improve outcomes compared with gemcitabine treatment [148–150], as seen with Cilengitide, an integrin ανβ 3/ανβ 5 inhibitor [151]. In contrast, a TGF-β receptor inhibitor showed promising results in a phase I trial combined with gemcitabine and nab-paclitaxel [152] (NCT02937272, NCT04158700). Galunisertib, another TGF- β receptor inhibitor, improved overall survival when combined with gemcitabine [153,154] (NCT02734160). However, another study has demonstrated therapeutic resistance through autotaxin, an enzyme produced by iCAFs, making autotaxin a promising and recently investigated target [155]. Targeting hyaluronan using a hyaluronidase (PEGPH20) failed to provide significant improvements in progression-free or overall survival [156,157] (NCT02715804). A phase I study combining defactinib, a FAK kinase inhibitor, with pembrolizumab (anti-PD-1) and gemcitabine showed some efficacy, and interestingly had an impact on the immunomodulation of the TME [158] (NCT02546531). Given the limited success of stromal depletion, novel approaches focusing on stromal normalization may generate better outcomes.

Ongoing clinical trials are focusing on reprogramming the PDAC TME to enhance therapeutic efficacy and antitumor immunity. Serplulimab, a novel anti-PD-1 monoclonal antibody, and metastasis-directed stereotactic body radiotherapy will be evaluated with standard gemcitabine plus nab-paclitaxel, aiming to improve overall survival for treatment-naive patients with recurrent or metastatic PDAC with a biologically rational ‘radio-immuno-chemotherapy’ strategy to overcome the immunologically cold and stromal-restricted TME that limits current outcomes [159] (NCT07336953). Another randomized phase 1 clinical trial is evaluating the CD73 inhibitor Quemliclustat, an enzyme that produces an immunosuppressive adenosine. Safety and efficacy of Quemliclustat combined with gemcitabine/nab-paclitaxel, with or without an anti-PD1, was evaluated in a phase 1b trial [160] (NCT04104672). Because of the vast evidence of the cross-talk between metabolism, ECM remodeling, and PDAC progression, some therapeutic strategies have targeted metabolism axis of PDAC. A combined regimen of different compounds targeting distinct aspects of cancer cell metabolism, including uptake of tyrosine in the TME and stimuli to increase oxidative stress, has been tested. However, only limited efficacy was observed [161].

More recently, enzymes linked to the biogenesis of fibrillar collagens such as lysyl oxidase and lysyl oxidases-like are investigated as ECM targets. Pan-lysyl oxidase inhibition, using the small-molecule inhibitor PXS-5505, decreases PDAC desmoplasia and stiffness, reduces invasion and metastasis, improves tumor perfusion, and enhances the efficacy of chemotherapy in patient-derived xenograft models [162]. The modulation of the Hippo pathway and YAP signaling has also been tested against PDAC ECM. The blockade of YAP and glutamine utilization effectively suppresses PDAC growth *in vivo*, and decreased ECM deposition signaling has also been investigated as a therapeutic approach to PDAC [163]. Furthermore, the blockade of the YAP1/TAZ-TEAD pathway enhanced KRAS^G12C^ inhibitors in KRAS^G12C^-resistant PDAC models [163,164]. Recently, a high-throughput screen of 36,000 compounds identified GNF-5, an Abelson tyrosine kinase inhibitor, as promising for modulating the myCAF phenotype and suggesting that reprogramming of CAF subtypes may be a therapeutic approach [165].

Beyond clinical trials, many different targets are being studied in preclinical models. Targeting glutamine transport and consequently YAP [163], FAP-positive cells like CAFs [166], semaphorins [167,168], glycoproteins [169], ROCK to remodel the stroma [170], and metalloproteinases [171–173] are just a few examples of the effort on developing a novel effective therapy for PDAC TME. Many other pathways are also being investigated as potential therapeutic targets. For example, chemotherapy resistance observed in organoids grown in stiffer matrices has been linked to increased expression of the efflux pump ABCG2, mediated by the CD44-hyaluronan axis. In addition, activating transcription factor 4 has been associated with gemcitabine, while the chloride intracellular channel 1/ROS/HIF1α pathway has promoted tumor growth by matrix stiffness stimuli [46,174,175].

## Conclusion

The PDAC TME is a highly complex and dynamic component that plays key physical and biochemical roles in PDAC development and progression. In recent years, advances in computational approaches and cancer models have improved our understanding of the PDAC TME, enabling the characterization of stromal and cellular heterogeneity, including CAFs, immune cells, and their influence on PDAC phenotypes. Thus, elucidating individual cellular populations and their spatial organization, remodeling dynamics, and interactions within the distinct TME niches is crucial to advance therapies for PDAC.

Despite these advances, translating this knowledge into clinical benefit remains very challenging. Innovative therapeutic approaches should target beyond stromal depletion, focusing on reprogramming the TME for a more therapy-responsive state, integrating these approaches with chemotherapy, immunotherapy, and other emerging targeted pathways. Finally, the development of relevant preclinical models that incorporate ECM composition, stiffness, and cellular-stromal interactions will be fundamental to identify innovative, efficacious, and personalized therapeutic strategies for PDAC.

## Perspectives

- PDAC is one of the deadliest cancers, and the complex TME is a main factor limiting the development of novel and efficient therapies.
- The PDAC TME is a key factor in tumor malignancy and progression, and understanding the tumor-stroma biology could lead to the identification of novel targets.
- To advance the development of therapeutic strategies that improve patient overall survival, it is necessary to comprehend and prioritize the role of TME in response to therapies. Thus, ECM components may become future novel targeted therapies.

## Open Access

Open access for this article is provided via our Subscribe to Open programme, made possible through the support of our subscribing institutions. Learn more here: https://portlandpress.com/pages/open_access_options_and_prices#conditional.

## Abbreviations

ABCG2: ATP-binding cassette sub-family G member 2
apCAFs: antigen-presenting fibroblasts
CAFs: cancer-associated fibroblasts
CCL19: C-C motif chemokine ligand 19
CCL21: C-C motif chemokine ligand 21
COL1: type I collagen
CSCs: cancer stem cells
CXCL12: CXC motif chemokine ligand 12
CXCR4: C-X-C chemokine receptor type 4
DDR1: discoidin domain receptor 1
E2F: early 2 factor
ECM: extracellular matrix
EMT: epithelial-mesenchymal transition
FAK: focal adhesion kinase
FAP: fibroblast-associated protein
FGA: fibrinogen A
FGB: fibrinogen B
FGG: fibrinogen G
FN1: fibronectin
FOLFIRINOX: leucovirin, fluorouracil, irinotecan and oxaliplatin
FOXP1: forkhead box P1
GM-CSF: granulocyte-macrophage colony-stimulating factor
H-SCAFs: high senescent CAFs
HIF-α: hypoxia-inducible factor 1-alpha
iCAFs: inflammatory CAFs
ICAM-1: intercellular adhesion molecule 1
IFN-γ: interferon gama
IGF1: insulin-like growth factor
IL-6: interleukin-6
kPa: kilopascal
KRAS: kristen rat sarcoma viral oncogene homolog
LOX: lysil oxidase enzymes
MAPK: mitogen-activated protein kinase
MDSC: myeloid-derived suppressor cells
meCAFs: metabolic state CAFs
MHC-II: major histocompatibility complex II
MMP3: matrix metalloproteinase-3
mTOR: mechanistic target of rapamycin
myCAFs: myofibroblasts
NE: norepinephrine
NET: neutrophil extracellular trap
NF-κβ: nuclear factor kappa B
NK cells: natural killer cells
NRF2: nuclear factor erythroid 2-related factor 2
OS: overall survival
PD1: programmed cell death protein 1
PDAC: pancreatic ductal adenocarcinoma
PEGPH20: pegvorhyaluronidase alfa
PFS: progression-free survival
PI3K: phosphoinositide 3-kinase
PIKfyve: phosphoinositide kinase, FYVE-type zinc finger containing11
PLOD: procollagen-lysine, 2-oxoglutarate 5-dioxygenase
PNI: perineural invasion
PSCs: pancreatic stellate cells
RB: retinoblastoma tumor suppressor gene
rCAFs: reticular CAFs
ROCK: Rho-associated, coiled-coil-containing protein kinase
ROS: ROS proto-oncogene 1, receptor tyrosine kinase
scRNA-seq: single-cell RNA sequencing
SLUG: snail family transcriptional repressor 2
SMAD 2/3/4: suppressor of mothers against decapentaplegic homolog 2,3,4
SNAIL: snail family transcriptional repressor
sub-TMEs: sub-tumor microenvironments
TAAs: tumor-associated antigens
TAN: tumor-associated neutrophils
tCAFs: tumor-like CAFs or transitional CAFs
TGF-β: transforming growth factor-β
Th17: T helper 17 cells
TME: tumor microenvironment
Tregs: regulatory T cells
TREM2: triggering receptor expressed on myeloid cells 2
TRPV: transient receptor potential vanilloid
VCAM-1: vascular cell adhesion molecule-1
VEGF: vascular endothelial growth factor
WNT: wingless-type MMTV integration site family, member 1
YAP: yes-associated protein
ZEB1/ZEB2: zinc finger E-box binding homeobox 1,2
